# The Infection of Physicians with Syphilis

**Published:** 1889-05

**Authors:** 


### *THE INFECTION OF PHYSICIANS WITH SYPHILIS.*

IN the *Centralblatt für Chirurgie*, 1889, No. 13, is a letter to members of the medical profession, in which the writer, Dr. S., asks their opinion upon the following question: "When can a physician, surgeon, obstetrician, etc., who has been infected with syphilis, again begin his practical work without fearing that his manipulations will infect his patients?"

Dr. S. quotes Fournier as saying that three years must elapse between the time of infection and marriage, and believes that the relation of physician and patient is much more intimate than that of a man to his wife in the marriage relation, inasmuch as the physician deals so often with fresh wound surfaces, ulcers, etc.

He can find nothing in literature upon the subject, hence his letter. He thinks the answer requires great caution, for it is much easier to forbid marriage than to keep a physician, who must support himself and family, away from his practice for a long time. The question is also raised as to what the chief physician of a hospital is to do when his subordinates (the assistants, nurses, midwives, etc.) become infected. The replies to these important questions will doubtless be read with much interest by physicians generally.

